# Homocysteine activates T cells by enhancing endoplasmic reticulum-mitochondria coupling and increasing mitochondrial respiration

**DOI:** 10.1007/s13238-016-0245-x

**Published:** 2016-02-08

**Authors:** Juan Feng, Silin Lü, Yanhong Ding, Ming Zheng, Xian Wang

**Affiliations:** Department of Physiology and Pathophysiology, School of Basic Medical Science, Peking University, Key Laboratory of Molecular Cardiovascular Science, Ministry of Education, Beijing, 100191 China

**Keywords:** homocysteine, T cell, mitochondria, endoplasmic reticulum stress

## Abstract

Hyperhomocysteinemia (HHcy) accelerates atherosclerosis by increasing proliferation and stimulating cytokine secretion in T cells. However, whether homocysteine (Hcy)-mediated T cell activation is associated with metabolic reprogramming is unclear. Here, our *in vivo* and *in vitro* studies showed that Hcy-stimulated splenic T-cell activation in mice was accompanied by increased levels of mitochondrial reactive oxygen species (ROS) and calcium, mitochondrial mass and respiration. Inhibiting mitochondrial ROS production and calcium signals or blocking mitochondrial respiration largely blunted Hcy-induced T-cell interferon **γ** (IFN-**γ**) secretion and proliferation. Hcy also enhanced endoplasmic reticulum (ER) stress in T cells, and inhibition of ER stress with 4-phenylbutyric acid blocked Hcy-induced T-cell activation. Mechanistically, Hcy increased ER-mitochondria coupling, and uncoupling ER-mitochondria by the microtubule inhibitor nocodazole attenuated Hcy-stimulated mitochondrial reprogramming, IFN-**γ** secretion and proliferation in T cells, suggesting that juxtaposition of ER and mitochondria is required for Hcy-promoted mitochondrial function and T-cell activation. In conclusion, Hcy promotes T-cell activation by increasing ER-mitochondria coupling and regulating metabolic reprogramming.

## INTRODUCTION

Homocysteine (Hcy) is a sulfur-containing non-constitutive amino acid derived from the essential amino acid methionine. Numerous clinical studies have established hyperhomocysteinemia (HHcy) as an independent risk factor for cardiovascular diseases in humans. We and others have demonstrated that HHcy accelerates atherosclerosis by affecting the immuno-inflammatory response, stimulating chemokine/cytokine secretion in monocytes and T cells, and repressing regulatory T-cell functions (Feng et al., 2009; Ma et al., 2013; Zeng et al., 2003; Zhang et al., 2002). Although reactive oxygen species (ROS) were identified as mediators in the process, the exact mechanism of Hcy-mediated lymphocyte activation is not well understood. Recent studies have revealed the intercrossing of pathways in the regulation of immune and metabolic systems (MacIver et al., 2013). Alterations in metabolism at both cellular and tissue levels affect specific T-cell functions (MacIver et al., 2013). However, whether the cellular metabolism is changed during Hcy-induced T-cell activation is unclear, and if changed, the underlying mechanism is unknown.

The endoplasmic reticulum (ER) and mitochondria are important cellular organelles in maintaining cell homeostasis, and dysfunction of each part is associated with multiple disorders, especially metabolic diseases. Emerging evidence showed that in addition to their distinct functions in regulating cellular processes, the ER and mitochondria interact with each other physically and functionally. The ER-mitochondria interaction enables the exchange of signals, lipids, and proteins (Szabadkai and Duchen, 2008). Enhancing ER-mitochondria coupling can increase mitochondrial calcium flux from the ER, thereby promoting mitochondrial respiration and adenosine triphosphate (ATP) production (Balaban, 2009; Brown, 1992). ER stress induced in HeLa cells increased ER-mitochondria coupling, thus elevating mitochondrial respiration and bioenergetics (Bravo et al., 2011). More recently, it was reported that chronic enrichment of hepatic ER-mitochondria coupling led to mitochondrial dysfunction in obesity (Arruda et al., 2014). It has been reported that ER and mitochondrial homeostasis play important and emerging functional roles respectively in T-cell development, innate immune responses, dendritic cell and macrophage functions (Bettigole and Glimcher, 2015; Cubillos-Ruiz et al., 2015; Martinon et al., 2010; Martinon and Glimcher, 2011; Staton et al., 2011). However, whether the coupling between the ER and mitochondria is involved in the regulation of Hcy-mediated T cell activation has not yet been studied.

In the present study, we explored the possible role of ER-mitochondria coupling and mitochondrial metabolism in Hcy-activated T cells. Our data showed that Hcy activates T cells through inducing ER stress. This subsequently enhances ER-mitochondria coupling and mitochondrial metabolic reprogramming which includes increased mitochondrial ROS production, calcium signal, membrane potential, and mass.

## RESULTS

### Reprogramming of mitochondrial metabolism in T cells from HHcy mice

**Figure 1:**
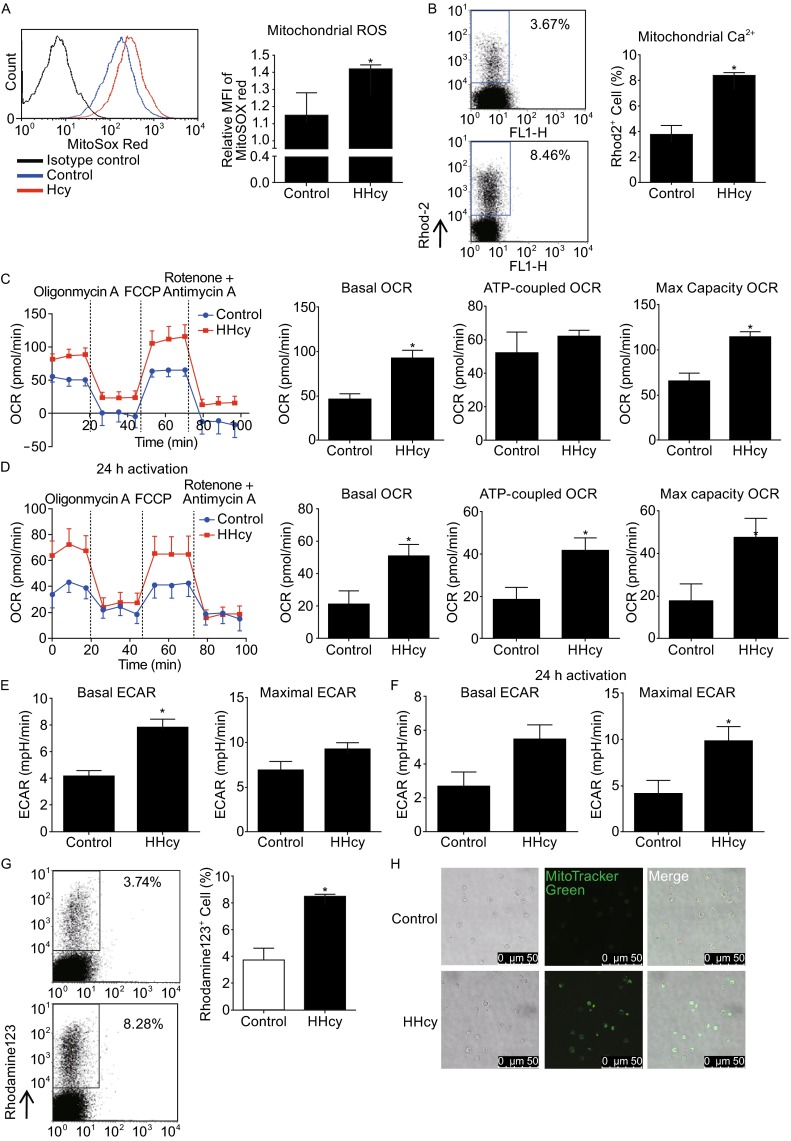
**Reprogramming of mitochondrial metabolism in T cells from HHcy mice**. Flow cytometry of splenic T cells from mice fed with or without Hcy and stained with MitoSOX Red (A) or Rhod-2 (B). Traces of OCR of splenic T cells from control or HHcy mice (C) or stimulated with anti-CD3 antibody for additional 24 h (D) as measured by the XF24 metabolic analyzer, with additions of mitochondrial effectors at time points indicated (left), and quantification of the basal OCR, max capacity OCR, and ATP-linked OCR (right). ECAR of splenic T cells from control or HHcy mice (E) or stimulated with anti-CD3 antibody for additional 24 h (F) as measured by the XF24 metabolic analyzer, and quantification of the basal and maximal ECAR. Flow cytometry of T cells stained with rhodamine 123 (G). (H) Confocol images of T cells loaded with MitoTracker Green. Results are mean ± SEM of six mice per group. *, *P* < 0.05 vs. control

Hcy treated mice were established to investigate the *in vivo* role of Hcy in regulating T cell mitochondrial metabolism. Our previous study found that ROS serves as a mediator in concanavalin A-activated T-cell proliferation potentiated by Hcy (Zhang et al., 2002). Here we further determined whether mitochondrial ROS was changed in response to Hcy stimulation. In consistent with our previous study (Zhang et al., 2002), the global ROS levels in T cells isolated from HHcy mice were increased (data not shown). HHcy also greatly increased mitochondrial ROS levels, as determined by the mitochondrial-specific superoxide anion probe MitoSOX Red, from 1.15 ± 0.13 mean fluorescence intensity [MFI] in cells from control mice to 1.39 ± 0.02 in cells from HHcy mice (Fig. 1A), indicating that increased mitochondrial ROS, the main source of ROS, may respond to HHcy-induced T-cell ROS production. This finding is in general agreement with a previous study in mice vaccinated with the lymphocytic choriomeningitis virus, in which authors found that mitochondrial ROS regulated T-cell activation and resulted in increased interleukin 2 production and antigen-specific expansion (Sena et al., 2013). We next examined the mitochondrial content of calcium, another important metabolism-associated mitochondrial signal, by loading T cells with the mitochondrial calcium probe Rhod-2. HHcy significantly increased Rhod-2 positive T cells to a 2.3-fold of control cells, from 3.67% ± 0.7% in control T cells vs. 8.46% ± 0.3% in HHcy treated cells (Fig. 1B). These results revealed that *in vivo* HHcy regulates both mitochondrial ROS and calcium signals in T cells.

Because mitochondrial ROS levels and calcium signals are closely linked with mitochondrial metabolism, we then determined mitochondrial oxidative phosphorylation in response to HHcy. T cells were sequentially treated with the ATP synthase inhibitor oligomycin, the uncoupler carbonylcyanltiep-trifluoro-methoxyphenylhydrazone (FCCP), and the combination of the electron-transport-chain inhibitor rotenone with antimycin A, and oxygen consumption rate (OCR), attributed to basal, ATP coupled, and maximal respiration, was monitored. HHcy increased both basal and maximal OCR, from 49 ± 8 pmol/min in control cells to 90 ± 12 pmol/min in HHcy treated cell for basal OCR and 70 ± 10 pmol/min to 120 ± 9 pmol/min for maximal OCR, while the ATP-coupled OCR was not altered, although with a slight trend of increase (Fig. 1C). To further confirm the effect of HHcy on T cell mitochondrial respiration, we also examined OCR in T cells isolated from HHcy mice in the presence of anti-CD3 antibody *in vitro* for additional 24 h. Likewise, the overall OCRs were increased, with basal OCR from 21 ± 11 pmol/min in control cells to 50 ± 10 pmol/min in HHcy treated cell, maximal OCR from 17 ± 12 pmol/min to 47 ± 14 pmol/min, and ATP-coupled OCR from 18 ± 13 pmol/min to 40 ± 11 pmol/min (Fig. 1D). Accompanying with the upregulated OCR, basal and maximal extracellular acidification rates (ECAR) were also increased by HHcy with (Fig. 1F) or without (Fig. 1E) anti-CD3 antibody *in vitro* for additional 24 h. Taken together, our results suggest that HHcy enhances mitochondrial respiration, probably through regulating mitochondrial ROS or calcium signals.

Increased mitochondrial respiration and metabolism are usually associated with increased mitochondrial membrane potential (Bravo et al., 2011), so we next detected the mitochondrial membrane potential in T cells from HHcy mice. Flow cytometry data showed that the mitochondrial membrane potential, as indicated by rhodamine 123 positive cells, were largely increased in T cells from HHcy mice as compared with the cells from control mice, from 3.74 ± 1.08 in control cells to 8.28 ± 0.03 in HHcy cells (Fig. 1G). Furthermore, staining of mitochondria probe MitoTracker Green showed that mitochondrial mass was significantly increased in HHcy-treated cells (Fig. 1H).

Collectively, our data from *in vivo* experiments showed that HHcy regulates mitochondrial metabolism through increasing mitochondrial ROS and calcium signals, and enhancing mitochondrial respiration, membrane potential, and mass.

### Hcy regulates mitochondrial metabolism in T cells *in vitro*

**Figure 2:**
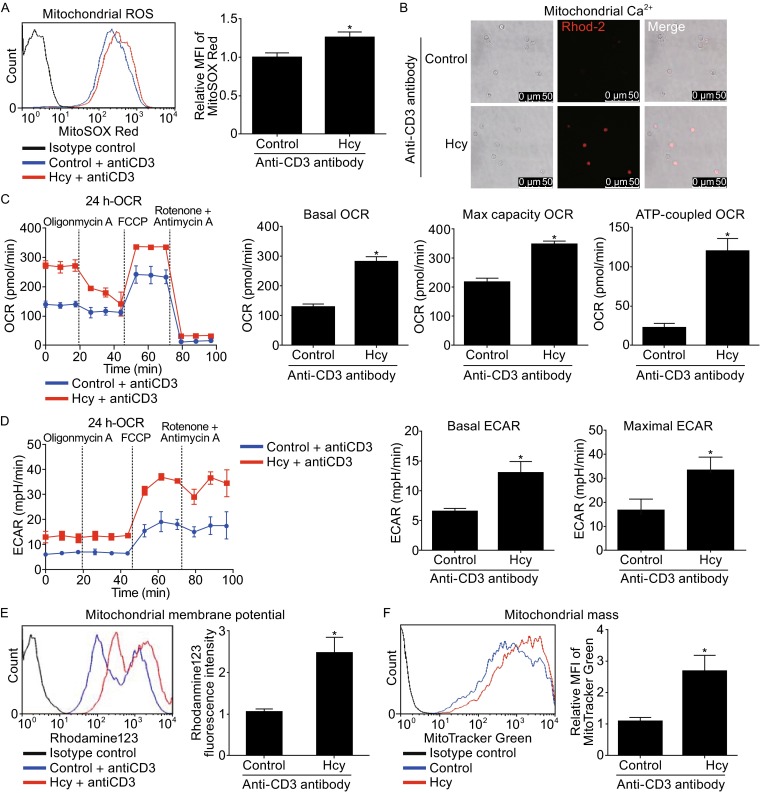
**Altered T-cell mitochondrial metabolism in response to Hcy stimulation**. T cells were incubated with or without Hcy (50 μmol/L) in the presence of anti-CD3 antibody for 24 h. (A) Flow cytometry of T cells stained with MitoSOX Red (left), and quantification (right). (B) Confocol images of T cells loaded with Rho-2. (C) Traces of OCR of T cells as measured by the XF24 metabolic analyzer, with additions of mitochondrial effectors at time points indicated (left), and quantification of the basal OCR, max capacity OCR, and ATP-linked OCR (right). (D) Traces of ECAR of T cells as measured by the XF24 metabolic analyzer, and quantification of the basal and maximal ECAR (right). Flow cytometry of T cells stained with rhodamine 123 (E) and MitoTracker Green (F). Data are mean ± SEM from 3 independent experiments. *, *P* < 0.05 vs. control

We further investigated the effect of Hcy in cultured T cells. Similarly, Hcy (50 μmol/L) stimulation for 24 h increased mitochondrial ROS levels, as indicated by the MitoSOX Red MFI, from 0.99 ± 0.02 in control to 1.26 ± 0.03 in Hcy-treated cells (Fig. 2A). Mitochondrial calcium content in cultured T cells was also increased by Hcy stimulation for 24 h, as showed by the increased fluorescent signals of Rhod-2 (Fig. 2B). Moreover, in consistent with our results from HHcy mice, Hcy increased OCR in cultured T cells, with the basal OCR from 120 ± 10 pmol/min in control cells to 274 ± 22 pmol/min in Hcy treated cells, maximal OCR from 223 ± 11 pmol/min to 351 ± 9 pmol/min, and ATP-coupled OCR from 25 ± 6 pmol/min to 123 ± 21 pmol/min (Fig. 2C). At the same time, basal and maximal ECAR were also upregulated by Hcy (Fig. 2D). Likewise, Hcy enhanced mitochondrial membrane potential, as indicated by rhodamine 123 fluorescence intensity, from 1.04 ± 0.08 in control T cells to 1.31 ± 0.07 in Hcy-treated cells (Fig. 2E), and increased mitochondrial mass, indicated by the MFI of MitoTracker Green, from 1.09 ± 0.04 in control to 1.30 ± 0.05 in Hcy-treated cells (Fig. 2F).

Therefore, both *in vivo* and *in vitro* data confirmed that Hcy regulates T cell mitochondrial metabolism, through increasing mitochondrial ROS production and calcium content, mitochondrial respiration, membrane potential, and mass.

### Mitochondrial metabolism reprogramming mediates Hcy-induced T cell activation

**Figure 3:**
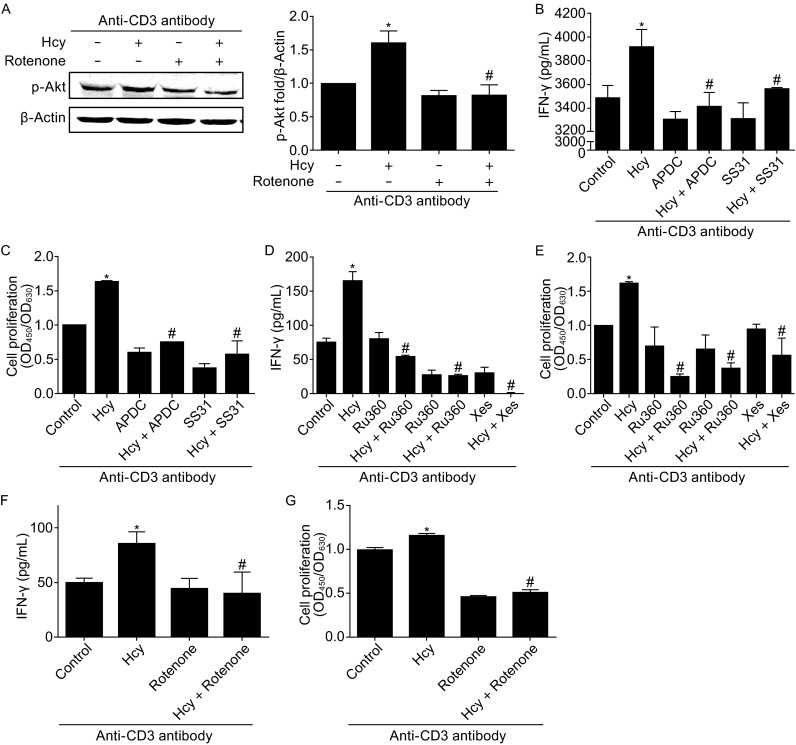
**Mitochondrial metabolism mediates Hcy-induced T cell activation**. (A) Western blot of phosphorylated Akt in T cells. (B) ELISA analysis of T-cell IFN-γ secretion in response to Hcy (50 μmol/L, 24 h) stimulation in the presence of mitochondrial ROS scavengers APDC and SS31; (C) T cell proliferation by CCK8 assay. T-cell IFN-γ secretion (D) or proliferation (E) in response to Hcy stimulation with or without the mitochondrial Ca^2+^ uniporter inhibitors Ru360 (10 μmol/L), RuRed (10 μmol/L) or IP3 receptor inhibitor Xestospongin C (Xes, 2 μmol/L). IFN-γ secretion (F) or cell proliferation (G) in response to Hcy with or without mitochondrial OXPHOS inhibitor rotenone (1 μmol/L). Data are mean ± SEM from 3 independent experiments. *, *P* < 0.05 vs. control. #, *P* < 0.05 vs. Hcy

Mitochondrial metabolism is closely involved in multiple cell processes in immune system (MacIver et al., 2013), so we sought to understand whether Hcy-altered mitochondrial metabolism contributed to Hcy-induced T-cell activation. We at first examined the Akt phosphorylation in response to Hcy stimulation, which indicates the activation of TCR/CD28 signal pathway. While Hcy (50 μmol/L, 24 h) increased the phosphorylation level of Akt in T cells to about 1.5-fold of the control cells, mitochondrial respiration chain inhibitor rotenone (20 nmol/L) totally abolished this Hcy-increased Akt phosphorlation to basal level (Fig. 3A), indicating a possible involvement of mitochondrial metabolism in T cell activation. We next explored if Hcy-mediated T cell mitochondrial metabolism was related to IFN-γ secretion and cell proliferation, two markers of T-cell activation (Feng et al., 2009). IFN-γ secretion by Hcy stimulation was increased to 112.65% ± 5.59% of that in control cells, and the Hcy-increased T-cell IFN-γ secretion was totally inhibited by ammonium 1-pyrrolidinedithiocarbamate (APDC; 100 μmol/L) or SS31 (50 μmol/L), two mitochondrial-specific ROS scavengers, to a level comparable to that in control cells (Fig. 3B). Similar results were obtained from cell proliferation experiments. Both APDC and SS31 completely inhibited Hcy-increased T-cell proliferation: fold of the control (OD_450_/OD_630_ absorbance) was decreased from 1.64 ± 0.00 in Hcy-treated cells to 0.75 ± 0.00 or 0.57 ± 0.14 in cells with APDC or SS31, respectively (Fig. 3C). Likewise, inhibition of mitochondrial calcium overload by the mitochondrial calcium uniporter inhibitors Ru360 (10 μmol/L) or RuRed (10 μmol/L), or by IP3 receptor inhibitor Xestospongin C (2 μmol/L), all abolished Hcy-increased IFN-γ secretion and proliferation (Fig. 3D and 3E). These results suggest that the increased mitochondrial ROS production and calcium signal participate in Hcy-mediated T-cell activation. Furthermore, inhibition of mitochondrial respiration by rotenone (20 nmol/L) greatly abolished Hcy-increased IFN-γ secretion from 80.71 ± 15.10 pg/mL in control cells to 40.81 ± 18.16 pg/mL in rotenone-treated cells (Fig. 3F), and cell proliferation from 1.15 ± 0.03 to 0.51 ± 0.03 fold of control (OD_450_/OD_630_ absorbance) in rotenone-treated cells (Fig. 3G). Therefore, our data here suggest that Hcy may activate T cells by reprogramming mitochondrial metabolism.

### Hcy enhances ER stress in T cells

**Figure 4:**
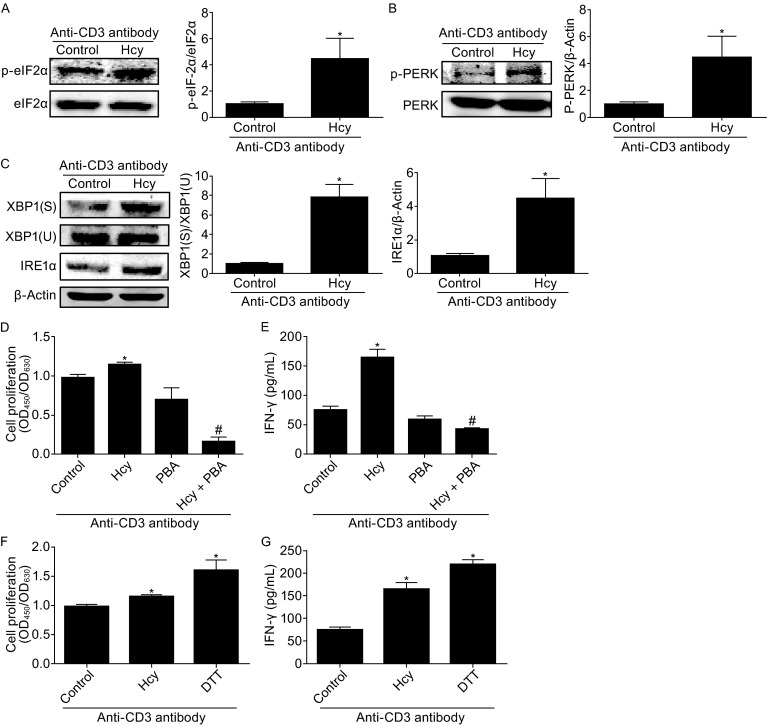
**Hcy enhances T cell ER stress**. T cells were incubated with or without Hcy (50 μmol/L) for 4 h with anti-CD3 antibody. Western blot analysis of phosphorylated eIF2α (A), phosphorylated PERK (B), and protein levels of IRE1α and spliced (S) and unspliced (U) XBP1 (C) in T cells with or without Hcy stimulation. β-Actin as a protein loading control. (D) CCK8 assay of cell proliferation with Hcy stimulation with or without ER stress inhibitor PBA or (F) ER stress stimulator DTT. ELISA of IFN-γ with Hcy stimulation with or without PBA (E) or DTT (G). Data are mean ± SEM from 3 independent experiments. *: *P* < 0.05 vs. Control. #, *P* < 0.05 vs. Hcy

The ER is physically and functionally linked with mitochondria (Bravo et al., 2011). Cellular stress stimuli including ER stress could lead to an oxidative stress response, which subsequently activates immune responses (Katika et al., 2011). We next investigated the possibility of whether Hcy-induced mitochondrial metabolic changes and T-cell activation are related to ER stress. We found that in cultured mouse splenic T cells, treatment with Hcy (50 μmol/L, 4 h) increased the protein levels of ER stress markers including p-eIF2a (Fig. 4A), p-PERK (Fig. 4B), IRE-1α, and spliced XBP-1 (Fig. 4C) to 4.0-, 4.4-, 7.2-, and 4.2-fold comparing with control cells. More importantly, inhibition of ER stress with PBA (5 mmol/L) almost completely abolished Hcy-induced T-cell proliferation and IFN-γ secretion (Fig. 4D and 4E), whereas stimulation of ER stress with dithiothreitol (DTT) (0.1 mmol/L) *per se* was sufficient to mimic Hcy-induced T-cell proliferation and IFN-γ secretion (Fig. 4F and 4G). Thus, our results suggest that ER stress might be an important player in Hcy-induced T-cell activation.

### Hcy reprograms mitochondrial metabolism and activates T cells by enhancing ER-mitochondria coupling

**Figure 5:**
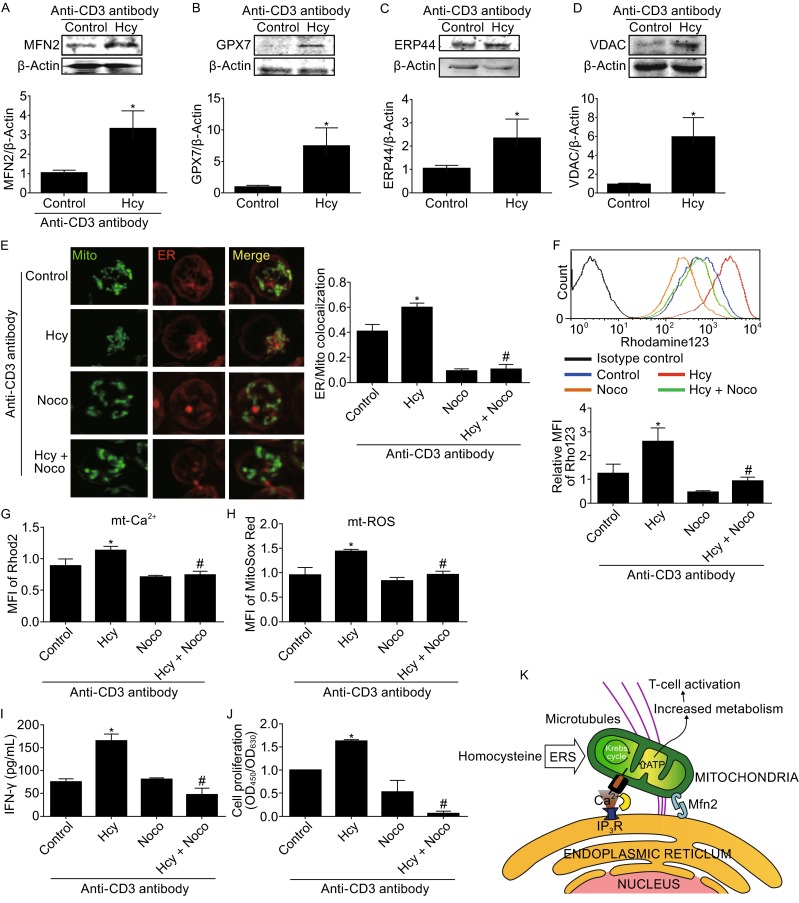
**Hcy reprograms mitochondrial metabolism and activates T cells through extending ER-mitochondria interaction**. Western blot analysis of MFN2 (A), GPX7 (B), ERP44 (C), and VDAC (D). (E) Structured illumination microscopy of ER-mitochondria colocalization (left) and quantification of the Manders’ coefficiency (right) of T cells with or without Hcy treatment for 4 h and nocodazole pretreated for one hour. (F), (G) and (H) MFI in T cells loaded with rhodamine 123, Rhod-2, and MitoSOX Red respectively, and quantified by FACS after Hcy stimulation for 24 h with or without nocodazole pretreatment. (I) IFN-γ secretion and (J) cell proliferation in response to Hcy with or without nocodazole pretreatment. (K) Proposed model of T-cell activation in response to Hcy stimulation. Data are mean ± SEM from 3 independent experiments. *, *P* < 0.05 vs. Control. #, *P* < 0.05 vs. Hcy

ER interacts with mitochondria, forming dynamic networks to induce metabolic responses (Bravo et al., 2011), so we wondered whether ER-mitochondria coupling is associated with Hcy-induced reprogramming of mitochondrial metabolism and T-cell activation. We first examined the levels of proteins at the mitochondria-associated membrane (MAM), including mitofusin 2 (MFN2), glutathione peroxidase 7 (Gpx7), ER resident protein 44 (ERP44), and voltage-dependent anion channel (VDAC), in response to Hcy stimulation. Hcy (50 μmol/L, 4 h) increased levels of MFN2 (Fig. 5A), Gpx7 (Fig. 5B), VDAC (Fig. 5D), and slightly increased ERP44 level (Fig. 5C) to 3.2-, 7.4-, 4.8-, and 2.2-fold of that in control cells. This indicates that Hcy enhances the coupling of mitochondria and ER.

To further visualize the interactions of mitochondria and ER, we used structured illumination microscopy (SIM) and found that the colocalization of ER and mitochondria was significantly increased with Hcy treatment for 4 h, from 0.41 ± 0.03 in control T cells to 0.60 ± 0.01 in Hcy-treated T cells (Fig. 5E). The ER and mitochondria are dynamic organelles, moving along the microtubule and interacting with each other (Bravo et al., 2011). In the present study, inhibition of microtubules by nocodazole (10 μmol/L) for one hour decreased the Hcy-induced ER and mitochondria colocalization, from 0.60 ± 0.01 in Hcy-treated T cells to 0.11 ± 0.02 in nocodazole-pretreated T cells (Fig. 5E). Taken together, our data suggest that Hcy increases ER-mitochondria interaction which requires microtubule-directed movement.

To determine whether the increased ER-mitochondria interaction contributes to the reprogramming of mitochondrial metabolism, we examined the effects of nocodazole on Hcy-mediated mitochondrial ROS level and calcium signal as well as mitochondrial membrane potential. Nocodazole greatly attenuated the Hcy-induced mitochondrial membrane potential, as indicated by relative rhodamine 123 MFI, from 2.60 ± 0.33 to 0.95 ± 0.10, comparable to the 1.26 ± 0.22 MFI in untreated control cells (Fig. 5F). Likewise, nocodazole (10 μmol/L) completely abolished Hcy-increased mitochondrial calcium signal to the level of control cells, as shown by Rhod 2 MFI (Fig. 5G). Moreover, nocodazole largely decreased Hcy-induced mitochondrial ROS level, as indicated by the changes of MitoSox Red fluorescence from 1.45 ± 0.01 MFI in Hcy-treated cells to 0.98 ± 0.04 in the presence of nocodazole pretreatment, whereas nocodazole alone did not alter T-cell mitochondrial ROS production as compared with control cells (Fig. 5H). Thus, the results suggest that Hcy-stimulated ER-mitochondria interaction contributes to the reprogrammed mitochondrial metabolism.

Finally, we investigated the effect of the ER-mitochondria interaction on Hcy-promoted T-cell activation. Hcy-activated IFN-γ secretion and cell proliferation were also significantly blocked with nocodazole (10 μmol/L) pretreatment, from 164.8 ± 14.36 to 49.27 ± 13.68 pg/mL for IFN-γ secretion and fold of control (OD_450_/OD_630_ absorbance) from 1.64 ± 0.01 to 0.07 ± 0.05 for cell proliferation (Fig. 5I and 5J). Therefore, the juxtaposition of mitochondria with the ER mediated by cytoskeleton microtubules is necessary for Hcy-promoted mitochondrial metabolic reprogramming and T-cell activation.

## DISCUSSION

In the present study, we have demonstrated for the first time that, both *in vivo* and *in vitro*, Hcy activates T cells by enhancing ER-mitochondria coupling, which subsequently reprograms mitochondrial metabolism by increasing mitochondrial ROS and calcium signal, membrane potential, mass, and oxygen consumption. Inhibiting these mitochondrial alterations completely blocks Hcy-induced T-cell activation. And disturbing ER-mitochondria coupling inhibits both Hcy-induced mitochondrial reprogramming and T-cell activation.

Lymphocytes and memory CD8^+^ T cells at different activation stages show distinct metabolic signatures, and metabolic programming is necessary for T-cell development (O’Sullivan et al., 2014). These evidences suggest that cell metabolism regulates T-cell function and cell fate in the immune system. We have previously reported that HHcy accelerates atherosclerosis by activating T cells (Feng et al., 2009; Ma et al., 2013; Zhang et al., 2002). Here we investigated whether metabolic reprogramming plays a critical role during Hcy-induced T-cell activation. Mitochondrial metabolism is indeed involved in T cell activation in response to Hcy stimulation, which is in general agreement with other studies of T cells in mice vaccinated with the lymphocytic choriomeningitis virus (Sena et al., 2013). Importantly, inhibiting mitochondrial changes by scavenging mitochondrial ROS, blocking mitochondrial calcium flux, or ceasing mitochondrial respiration, all abolished Hcy-induced T-cell proliferation and IFN-γ secretion, further supporting our hypothesis that Hcy regulates T-cell activation by reprogramming mitochondrial metabolism.

Emerging evidence highlights that T-cell activation leads to marked shifts in cell metabolism to protect against pathogens and to orchestrate the action of other immune cells (MacI et al., 2013). Quiescent T cells require predominantly ATP-generating processes, whereas proliferating effector T cells require high metabolic flux through growth-promoting pathways (MacI et al., 2013). Our preliminary data showed that both oxidative phosphorylation and aerobic glycolysis in T cells were upregulated with Hcy treatment (Figs. 1C–F, 2C, and 2D). However, whether Hcy modulates bioenergetic shift remains unclear. Here, our *in vivo* and *in vitro* data showed that Hcy largely increases overall T cell oxygen consumption rate, and inhibition of mitochondrial respiration totally blocked Hcy-mediated T cell activation, suggesting that Hcy-induced bioenergenic process participates in the T-cell activation. It has been reported that different T-cell subsets achieve specific functions through distinct energetic and biosynthetic pathways, and cell metabolic changes at celluar and systemic levels may enhance or suppress specific T-cell functions (Gerriets et al., 2015; MacI et al., 2013). We have previously reported the acceleration of atherosclerosis by HHcy via activating proinflammatory T cells and repressing regulatory T-cell function (Feng et al., 2009). How different metabolic modes of Hcy treatment on T-cell subtypes merits further determination.

ER is physically and functionally linked with mitochondria (Bravo et al., 2011). In addition to the finding that Hcy reprograms mitochondrial metabolism, we found that Hcy also triggers ER stress in T cells, and inhibition of ER stress blocks Hcy-activated T cells, while stimulation of ER stress mimics this activation, suggesting that Hcy activates T cells by triggering ER stress. These results agree with the findings in vascular endothelial cells (Hossain et al., 2003), hepatocytes (Yang et al., 2014), neurons (Wei et al., 2014), and adipocytes (Li et al., 2013), where Hcy also causes ER stress. ER interacts with mitochondria forming dynamic networks across all cells to regulate multiple cellular processes (Bravo et al., 2011). Our present study found that ER-mitochondria coupling in response to Hcy stimulation increased, as shown by upregulated ER-mitochondria proteins MFN2, Gpx7, ERP44, and VDAC. Since maintenance of proper MAMs and Ca^2+^ flux is essential for mitochondrial function and metabolic homeostasis, increased MAM formation may represent a generalized and early response to ER stress.

ER calcium channel IP3R resides on MAM site, in close proximity to the outer mitochondrial membrane. The increased ER-mitochondria coupling by Hcy may facilitate the uptake of Ca^2+^ from ER into the mitochondrial matrix through the low-affinity mitochondrial Ca^2+^ uniporter (MCU) (Arruda et al., 2014). Our present study found that T-cell activation was mediated by abnormal Ca^2+^ transport from the ER to mitochondria. Inhibition of mitochondrial Ca^2+^ overload by the mitochondrial MCU inhibitors Ru360 and RuRed or IP3 receptor inhibitor Xestospongin C all abolished Hcy-increased IFN-γ secretion and T-cell proliferation. Moreover, we found that inhibition of the Hcy-enhanced ER-mitochondria interaction by dissembling microtubules blocks mitochondrial calcium flux from the ER and decreases mitochondrial ROS levels and membrane potential. Functionally, inhibiting ER-mitochondria coupling by nocodazole diminished Hcy-induced T-cell activation. These findings suggest a working diagram that, the microtubule-directed increase of ER-mitochondria coupling as an early adaptive response to Hcy-mediated ERS, drives Ca^2+^ accumulation in the mitochondria that, in turn, leads to mitochondrial reprogramming, increased ROS generation, metabolism, and T-cell activation (Fig. 5K). Thus, it is reasonable to speculate that tightening of the ER-mitochondria interface, as an earlier event rather than a consequence of mitochondrial metabolic changes, may be responsible for promoting adaptive mitochondrial responses induced by Hcy.

Although the immune system has a critical role in protecting against diseases, energy demands must be finely regulated. We are beginning to understand how pathways that regulate early T-cell activation induced by Hcy are linked to those regulating metabolism. Because of the fundamental importance of cellular bioenergetics control in normal cellular physiology and pathophysiology, as well as the role of T-cell activation in diverse human pathologies, identification of this early process has broad implications. Manipulation of metabolic programs at an early stage may offer therapeutic opportunities for altering the immune responses in diseases. We have reported that HHcy promotes insulin resistance by inducing ER stress and inflammation in the adipocytes (Li et al., 2013). And a recent study by other groups revealed that obesity in mice led to tightened ER-mitochondria contact and increased mitochondrial oxidative stress and impaired metabolic homeostasis in the liver (Arruda et al., 2014). Therefore, our present study may have implications beyond Hcy-induced T-cell activation.

Taken together, our findings have revealed for the first time that the metabolic axis, consisting of ER stress, ER-mitochondria coupling, and mitochondrial metabolism, is involved in the Hcy-promoted T-cell activation. This work provides supporting evidence for an important role of mitochondrial metabolism in regulating early activation of T cells, and sheds new light on understanding the mechanisms underlying the pathogenesis of HHcy-accelerated metabolic syndrome.

## MATERIALS AND METHODS

### Animal and treatment

Six-week-old female C57BL/6J mice were provided by the Animal Center of Peking University Health Science Center (Beijing, China). Mice were fed normal mouse chow supplemented with or without 1.8 g/L D.L-Hcy (Sigma Chemical Co., St. Louis, MO) in drinking water (*n* = 6 for each) for 2 weeks, as we previously reported (Dai et al., 2006) with some modifications. The protocol was approved by the Committee on the Ethics of Animal Experiments of the Health Science Center of Peking University. All animal studies were performed in compliance with the U.S. Department of Health and Human Services Guide for the Care and Use of Laboratory Animals.

### **T-cell proliferation and IFN**-γ **production**

T cells were isolated and cultured as previously reported (Feng et al., 2009). The anti-CD3 antibody (1 µg/mL) was pre-coated onto all microplates overnight. Then T cells at 3 × 10^5^ cells/well in the anti-CD3-antibody-precoated microplates, with or without Hcy (50 µmol/L) for 4 h were used for evaluating ERS, or for 24 h were used for proliferation assay. Inhibitors were added one hour before the addition of Hcy. CCK8 (20 μL/well, DOJINDO, Kyushu Island, Japan) was added 6 h before analysis, and experiments were performed in triplicate. The absorbance was determined at 450-nm wavelength with a reference wavelength of 630 nm by use of a microplate reader (Bio-Rad Laboratories, Hercules, CA, USA). IFN-γ in the cell supernatant was measured by ELISA (NeoBioscience Technology, Shenzhen, China).

### Flow cytometry

T cells were stained with MitoTracker green (100 nmol/L), Fluo-3 (1 mmol/L), Rhod-2 (5 mmol/L) for 30 min or with Brefeldin A (5 µg/mL) for 5 h, then analyzed by FACS flow cytometry with Cell QuestPro software (BD Biosciences, USA).

### Western blot analysis

The protocol of Western blot analysis was as previously reported (Feng et al., 2009) with some modifications of indicated primary antibodies.

### Metabolism assays

Oxygen consumption rates (OCR) and extracellular acidification rates (ECAR) were measured using the XF-24 Extracellular Flux Analyzer (Seahorse Bioscience). The protocol was as reported (van der Windt et al., 2012) with some modifications. In total, 6 × 10^5^ cells with or without Hcy treatment for 24 h were seeded per well in 24-well XF microplates coated with Poly-L-lysine hydrobromide. Cell culture medium was replaced with XF Assay Medium supplemented with 11 mmol/L glucose and 1 mmol/L pyruvate and incubated in a CO_2_-free incubator at 37°C for 1 h for temperature and pH equilibration. The baseline oxygen consumption rate (OCR) was measured, then wells were injected sequentially with oligomycin (1 µmol/L) to measure the ATP-link OCR, oxidative phosphorylation uncoupler carbonylcyanltiep-trifluoro-methoxyphenylhydrazone (FCCP, 1 µmol/L) to determine maximal respiration, and rotenone (1 µmol/L) and antimycin A (1 µmol/L) to determine non-mitochondrial respiration. Experiments were performed with 3 or 4 wells of each plate used as technical replicates, and each experiment had at least three replicates. OCR was normalized by the amount of cells in each well.

For cell glycolysis measurement, T cells seeded in XF24 microplates as described above were cultured in XF base medium supplemented with 2 mmol/L L-glutamine in CO_2_-free incubator for 1 h. After three baseline ECAR measurements, cells were added sequentially with glucose (10 mmol/L) to measure the glucose metabolism, oligomycin (1 µmol/L) to measure the glycolytic capacity, and 2-deoxy-D-glucose (2-DG) (100 mmol/L) to measure the non-glycolytic acidification. Experimental treatments were performed on 3 or 4 wells of each plate as technical replicates and each experiment had at least 3 biological replicates. ECAR was normalized by the amount of protein in each well.

### Confocal microscopy

T cells were loaded with Rhod-2 in HBSS buffer supplemented with 0.02% pluronic acid F-127 for 30 min at 37°C, washed with phosphate buffered saline (PBS) buffer 3 times, and then incubated in the PBS buffer for 2 h at room temperature to allow for complete deesterification. Fluorescence was visualized by confocal microscopy with a 488-nm laser line. For mitochondrial network and ER colocalization, T cells were loaded with MitoTrack Green and ER-Tracker™ Red. One focal plane was analyzed. The images obtained were deconvolved and the background was subtracted by use of ImageJ. Colocalization between organelles was quantified by use of the Manders’ algorithm as described (Costes et al., 2004).

### Reagents and antibodies

D.L-Hcy, nocodazole, ammonium 1-pyrrolidinedithiocarbamate (APDC), and 4-phenylbutyric acid (PBA), and anti-voltage-dependent anion channel (VDAC) antibody were from Sigma Chemical Co. Anti-X-box binding protein 1 (anti-XBP-1) antibody was from Santa Cruz Biotechnology. Anti-inositol-requiring enzyme 1α (anti-IRE1α), anti-p-PKR-like endoplasmic reticulum kinase (anti-p-PERK), and anti-p-eukaryotic initiation factor 2 (anti-p-eIF2α) antibodies were from Cell Signal Technology. MitoTracker Green, Rhod-2, ER-Tracker™ Red, and MitoSOX Red were from Invitrogen. Anti-mitofusin 2 (anti-MFN2) antibody was from ABGENT. APC-IFN-γ was from BD Pharmagen. Brefeldin A (BFA) was from Biolegend. Anti-endoplasmic reticulum resident protein 44 (anti-ERP44) and anti-glutathione peroxidase 7 (anti-Gpx7) antibodies were gifts from Prof. Chih-chen Wang (Institute of Biophysics, Chinese Academy of Sciences, Beijing).

### Statistical analysis

All data are reported as mean ± SEM. Data were analyzed by use of the GraphPad Prism software. Statistical analysis involved one-way ANOVA followed by the Student-Newman-Keuls test for multiple comparisons and Student’s unpaired *t*-test for comparisons between two groups. *P* < 0.05 was considered statistically significant.

## References

[CR1] Arruda AP, Pers BM, Parlakgül G, Güney E, Inouye K, Hotamisligil GS (2014). Chronic enrichment of hepatic endoplasmic reticulum-mitochondria contact leads to mitochondrial dysfunction in obesity. Nat Med.

[CR2] Balaban RS (2009). The role of Ca(2+) signaling in the coordination of mitochondrial ATP production with cardiac work. Biochim Biophys Acta.

[CR3] Bettigole SE, Glimcher LH (2015). Endoplasmic reticulum stress in immunity. Annu Rev Immunol.

[CR4] Bravo R, Vicencio JM, Parra V, Troncoso R, Munoz JP, Bui M, Quiroga C, Rodriguez AE, Verdejo HE, Ferreira J, Iglewski M, Chiong M, Simmen T, Zorzano A, Hill JA, Rothermel BA, Szabadkai G, Lavandero S (2011). Increased ER-mitochondrial coupling promotes mitochondrial respiration and bioenergetics during early phases of ER stress. J Cell Sci.

[CR5] Brown GC (1992). Control of respiration and ATP synthesis in mammalian mitochondria and cells. Biochem J.

[CR6] Costes SV, Daelemans D, Cho EH, Dobbin Z, Pavlakis G, Lockett S (2004). Automatic and quantitative measurement of protein-protein colocalization in live cells. Biophys J.

[CR7] Cubillos-Ruiz JR, Silberman PC, Rutkowski MR, Chopra S, Perales-Puchalt A, Song M, Zhang S, Bettigole SE, Gupta D, Holcomb K, Ellenson LH, Caputo T, Lee AH, Conejo-Garcia JR, Glimcher LH (2015). ER stress sensor XBP1 controls anti-tumor immunity by disrupting dendritic cell homeostasis. Cell.

[CR8] Dai J, Li W, Chang L, Zhang Z, Tang C, Wang N, Zhu Y, Wang X (2006). Role of redox factor-1 in hyperhomocysteinemia-accelerated atherosclerosis. Free Radic Biol Med.

[CR9] Feng J, Zhang Z, Kong W, Liu B, Xu Q, Wang X (2009). Regulatory T cells ameliorate hyperhomocysteinaemia-accelerated atherosclerosis in apoE-/- mice. Cardiovasc Res.

[CR10] Gerriets VA, Kishton RJ, Nichols AG, Macintyre AN, Inoue M, Ilkayeva O, Winter PS, Liu X, Priyadharshini B, Slawinska ME, Haeberli L, Huck C, Turka LA, Wood KC, Hale LP, Smith PA, Schneider MA, MacIver NJ, Locasale JW, Newgard CB, Shinohara ML, Rathmell JC (2015). Metabolic programming and PDHK1 control CD4+ T cell subsets and inflammation. J Clin Invest.

[CR11] Hossain GS, van Thienen JV, Werstuck GH, Zhou J, Sood SK, Dickhout JG, de Koning AB, Tang D, Wu D, Falk E, Poddar R, Jacobsen DW, Zhang K, Kaufman RJ, Austin RC (2003). TDAG51 is induced by homocysteine, promotes detachment-mediated programmed cell death, and contributes to the cevelopment of atherosclerosis in hyperhomocysteinemia. J Biol Chem.

[CR12] Katika MR, Hendriksen PJ, van Loveren H, Peijnenburg A (2011). Exposure of Jurkat cells to bis (tri-n-butyltin) oxide (TBTO) induces transcriptomics changes indicative for ER- and oxidative stress, T cell activation and apoptosis. Toxicol Appl Pharmacol.

[CR13] Li Y, Zhang H, Jiang C, Xu M, Pang Y, Feng J, Xiang X, Kong W, Xu G, Li Y, Wang X (2013). Hyperhomocysteinemia Promotes Insulin Resistance by Inducing Endoplasmic Reticulum Stress in Adipose Tissue. J Biol Chem.

[CR14] Ma K, Lv S, Liu B, Liu Z, Luo Y, Kong W, Xu Q, Feng J, Wang X (2013). CTLA4-IgG ameliorates homocysteine-accelerated atherosclerosis by inhibiting T-cell overactivation in apoE(-/-) mice. Cardiovasc Res.

[CR15] MacIver NJ, Michalek RD, Rathmell JC (2013). Metabolic regulation of T lymphocytes. Annu Rev Immunol.

[CR16] Martinon F, Glimcher LH (2011). Regulation of innate immunity by signaling pathways emerging from the endoplasmic reticulum. Curr Opin Immunol.

[CR17] Martinon F, Chen X, Lee AH, Glimcher LH (2010). TLR activation of the transcription factor XBP1 regulates innate immune responses in macrophages. Nat Immunol.

[CR18] O’Sullivan D, van der Windt GJ, Huang SC, Curtis JD, Chang CH, Buck MD, Qiu J, Smith AM, Lam WY, DiPlato LM, Hsu FF, Birnbaum MJ, Pearce EJ, Pearce EL (2014). Memory CD8(+) T cells use cell-intrinsic lipolysis to support the metabolic programming necessary for development. Immunity.

[CR19] Sena LA, Li S, Jairaman A, Prakriya M, Ezponda T, Hildeman DA, Wang CR, Schumacker PT, Licht JD, Perlman H, Bryce PJ, Chandel NS (2013). Mitochondria are required for antigen-specific T cell activation through reactive oxygen species signaling. Immunity.

[CR20] Staton TL, Lazarevic V, Jones DC, Lanser AJ, Takagi T, Ishii S, Glimcher LH (2011). Dampening of death pathways by schnurri-2 is essential for T-cell development. Nature.

[CR21] Szabadkai G, Duchen MR (2008). Mitochondria: the hub of cellular Ca2+ signaling. Physiology (Bethesda).

[CR22] van der Windt GJ, Everts B, Chang CH, Curtis JD, Freitas TC, Amiel E, Pearce EJ, Pearce EL (2012). Mitochondrial respiratory capacity is a critical regulator of CD8+ T cell memory development. Immunity.

[CR23] Wei HJ, Xu JH, Li MH, Tang JP, Zou W, Zhang P, Wang L, Wang CY, Tang XQ (2014). Hydrogen sulfide inhibits homocysteine-induced endoplasmic reticulum stress and neuronal apoptosis in rat hippocampus via upregulation of the BDNF-TrkB pathway. Acta Pharmacol Sin.

[CR24] Yang X, Xu H, Hao Y, Zhao L, Cai X, Tian J, Zhang M, Han X, Ma S, Cao J, Jiang Y (2014). Endoplasmic reticulum oxidoreductin 1α mediates hepatic endoplasmic reticulum stress in homocysteine-induced atherosclerosis. Acta Biochim Biophys Sin (Shanghai).

[CR25] Zeng X, Dai J, Remick DG, Wang X (2003). Homocysteine mediated expression and secretion of monocyte chemoattractant protein-1 and interleukin-8 in human monocytes. Circ Res.

[CR26] Zhang Q, Zeng X, Guo J, Wang X (2002). Oxidant stress mechanism of homocysteine potentiating Con A-induced proliferation in murine splenic T lymphocytes. Cardiovasc Res.

